# The Impact of Scenarios on the Performance of Entrepreneurial Imaginativeness: Evidence From an Experiment

**DOI:** 10.3389/fpsyg.2022.813657

**Published:** 2022-03-31

**Authors:** Yang Chen, Min Wang, Yawen Liu, Ruoyu Lu

**Affiliations:** ^1^School of Management and Economics, University of Electronic Science and Technology of China, Chengdu, China; ^2^Institute of Electronic and Information Engineering of UESTC in Guangdong, Dongguan, China

**Keywords:** scenario, entrepreneurial imaginativeness, new venture ideas, creativity, imagination

## Abstract

With the advent of the era of artificial intelligence, “scenario” frequently appears in new product development and has gradually become an effective tool for analyzing user needs. However, the reasons for this phenomenon have not been explored in depth. New product development is a creative activity that requires product designers to imagine how people will live in the near future. So, we speculated that a familiar scenario that matches designers’ background (including knowledge, expertise, and experience) can spark their entrepreneurial imaginativeness by empathic simulation and conducted an experiment to research the impact of scenarios on the performance of entrepreneurial imaginativeness. Results of this study confirmed that a familiar scenario did indeed inspire entrepreneurial imaginativeness more than an unfamiliar scenario, especially for high entrepreneurial imaginativeness. This study provided a new respective for understanding the relationship between the empathy process and entrepreneurial opportunity recognition and evaluation processes and had practical implications for entrepreneurial practice, especially those that make human life better based on new digital technologies. Finally, we gave some suggestions on enhancing individuals’ entrepreneurial imaginativeness through different familiar scenarios and improving the team performance on creative tasks.

## Introduction

The term “scenario” was first introduced into business practice in the 1960s by the futurist Herman Kahn (Kahn and Wiener, 1967). Later, scenario analysis, as a planning method, was used by enterprises operating in unstable political and social environments (such as Royal Dutch/Shell Group, General Electric, and Lockheed) to predict possible future developments through hypothetical sequences of events (Gausemeier et al., 1998). With the advent of the era of artificial intelligence, “scenario” frequently appears at the product level (more micro than the organizational level) and is becoming an effective tool gradually for analyzing user needs. Through the lens of scenario, product developers can see possible future needs of people in some scenarios and propose different product concepts to address these needs (Passey et al., 2006). For instance, in October 2021, Meta CEO Mark Zuckerberg presented several product concepts for different scenarios in Meta’s metaverse (e.g., Horizon Home, for home spaces; Horizon Worlds, to create worlds or games; and Horizon Workrooms, for working) (López-Díez, 2021). Pillan et al. (2014) similarly confirmed that “scenarios presented by video can visualize implicit needs and fertile cues for designers” and “video-scenarios as a tool to support imagination can orient designers’ efforts toward an optimal solution in terms of human satisfaction.” In addition, the scenario method also links product development to the visions of sustainability at the societal level by a systemic view (Gaziulusoy et al., 2013). Therefore, the scenario method plays an important role in the early parts of new product development—idea generation, product conceptualization, and concept selection (Crawford, 2008).

Why are scenarios becoming the essential condition of business ideas generation? Perhaps, related psychology research can provide inspiration for answering this question. Packard and Burnham (2021) proposed a “simulated empathy theory” that connects the empathy process to entrepreneurial opportunity recognition and evaluation processes. This theory is used to help entrepreneurs to predict consumer demand before it exists, thereby seizing entrepreneurial opportunities. It is worth noting that empathy, as a type of vicarious imagination (Coplan, 2011), is “a rational imagination process, intentional and knowledge-based”; and empathic simulation, as a vicarious mental simulation, is “the imaginative play-out of a particular scenario through the empathizer’s empathic model” (Packard and Burnham, 2021). That is, the empathizer’s vicarious imagination is activated by a particular scenario that provides a specific space–time context for empathy simulation. In addition, a particular scenario also provides a specific space–time context for the application of the transcendental schema (Kant, 1908) that can be used to interpret present sensations acquired through empathy and give solutions to address these sensations.

However, as a kind of external factor directly acting on the imaginative process, what effects do scenarios have on the exertion of entrepreneurial imaginativeness? This seems to be an interesting and new research topic that has hardly been covered in previous studies. After reviewing the relevant literature on imagination, we got some clues. There are two kinds of imaginative processes, namely, “one that is unconscious, uncontrolled, and effortless, and another that is conscious, controlled, and effortful”; the performance of any given thought experiment relies more or less on one of two kinds of imaginative processes, depending on the performer’s abilities and experience (Stuart, 2021). Similarly, Kier and McMullen (2018) argued that an individual’s domain knowledge, expertise, and experience on innovation, communication, and administration are preconditions of entrepreneurial imaginativeness exerting. Therefore, we speculated that the fit between an individual’s background (including knowledge, expertise, and experience) and scenarios affects the performance of entrepreneurial imaginativeness and proposed Hypothesis 1 as the performance of entrepreneurial imaginativeness in a familiar scenario is better than in an unfamiliar scenario. In addition, entrepreneurial imaginativeness used to generate and select new venture ideas involves creative, social, and practical imaginativeness (Kier and McMullen, 2018), which have individual differences. People with a high level of creative, social, and practical imaginativeness generate a larger set of new venture ideas, presumably due to “their ability to make novel connections between seemingly unrelated things,” “their ability to translate the thoughts, feelings, and intentions of others into new venture ideas,” and “their ability to identify problems in need of solutions” (Kier and McMullen, 2018). So, we speculated that familiar scenarios enable high entrepreneurial imaginativeness to be more fully inspired than unfamiliar ones and proposed Hypothesis 2a to performers with a high level of entrepreneurial imaginativeness, their performance of entrepreneurial imaginativeness in a familiar scenario is better than that in an unfamiliar scenario. But entrepreneurial imaginativeness, as a cognitive skill, is relatively stable and difficult to significantly improve in a short time. Even supported by familiar scenarios, low entrepreneurial imaginativeness can hardly perform well. Therefore, we proposed Hypothesis 2b to performers with a low level of entrepreneurial imaginativeness, their performance of entrepreneurial imaginativeness in a familiar scenario is not significantly different from that in an unfamiliar scenario.

To empirically test our hypothesis, in this article, “scenario” was designed as an exogenous manipulated variable that affects the performance of entrepreneurial imaginativeness by matching with an individual’s background (including knowledge, expertise, and experience). We conducted an experiment in which 249 MBA students were randomly divided into three groups to conceive new venture ideas based on Fifth Generation Mobile Communication (5G) Technology in three scenario settings, namely, “smart city” (a scenario relatively familiar to the public), “smart factory” (a scenario relatively unfamiliar to the public), and “no-scenario” (compared with the other two scenarios). This enabled the manipulation of scenarios as familiar or unfamiliar while comparing this manipulation with the no-scenario group. Furthermore, drawing on the mature scale of Kier and McMullen (2018) to measure entrepreneurial imaginativeness, we distinguished participants with different levels of entrepreneurial imaginativeness to test the influences of three scenario settings on their performance of entrepreneurial imaginativeness.

## Methodology

### Participants and Design

A total of 249 Master of Business Administration (MBA) students participated in Study 1 in exchange for a nice present, which ensured participants had basic business operations knowledge and experience. Using a between-group experimental design, participants were randomly assigned to a (1) smart city (85 people), (2) smart factory (86 people), or (3) no-scenario (78 people). Finally, 219 valid samples were collected, including 75 in the smart city group, 71 in the smart factory group, and 73 in the no-scenario group.

A total of 219 participants with ages ranging from 21 to 55 years (M_*age*_ = 32.89, SD_*age*_ = 4.485) were included; 48.4% were men and 99.5% had a bachelor’s degree or above. All the participants came from 20 different industries, with 85.4% having more than 5 years of work experience (M_*working*–years_ = 5.13, SD_*working*–years_ = 1.114); 38 (17.14% of the total) had tried to start a business once in their career; 26 (11.9% of the total) tried two times to start a business, and 5 (2.3% of the total) tried three or more times.

### Materials and Measures

#### Scenario

A particular scenario that inspires the empathizer’s vicarious imagination entails “the who, what, when, where, why, and how—the happenings within that experience, the contextual factors surrounding it, and the motivations and mechanisms that underpin it” (Packard and Burnham, 2021). In addition, Koh and Leung (2019) confirmed that a future orientation, as a means, can facilitate creativity through activating change and progress schemas to broaden thinking. Thus, both the familiar and unfamiliar scenarios chosen by us are future-orientated and equipped with the characteristics listed in Table 1. Based on the above criteria, we chose the smart city as the familiar scenario and the smart factory as the unfamiliar scenario and showed participants both scenarios through video. Specifically, a smart city involves almost all aspects of social life, such as government affairs, transportation, medical care, education, culture, entertainment, and environmental protection. Most of the public has an intuitive and specific understanding of these elements, processes, and existing problems. However, smart factory involves professional knowledge and industry experience, such as cognitive automation, cyber-physical system-based real-time monitoring (Stehel et al., 2021), sustainable manufacturing Internet of Things (Hawkins, 2021), and industrial big data analytics (Kovacova and Lewis, 2021). These are unfamiliar for most of the practitioners in the non-manufacturing and processing industries.

**TABLE 1:** Characteristics of a familiar/unfamiliar scenario.

A familiar scenario	An unfamiliar scenario
(1) The attributes, features, and performance of elements in this scenario are known well to most public	(1) The attributes, features, and performance of elements in this scenario are known less to most public
(2) It is not a hard work for most public to identify the similarity, differences, and relevance among the elements in this scenario accurately	(2) It is a hard work for most public to identify the similarity, differences, and relevance among the elements in this scenario accurately
(3) Most public had or are having the same or similar experience as the description in this scenario	(3) Most public don’t have the same or similar experience as the description in this scenario
(4) It is easy for most public to take the perspective of others to experience others’ irritations, annoyances, and frustrations with existing products or services in this scenario	(4) Most public have little opportunity to take the perspective of others to experience others’ irritations, annoyances, and frustrations with existing products or services in this scenario
(5) The same or similar professional knowledge, methods, and procedures as what is needed in this scenario are possessed by most public	(5) The same or similar professional knowledge, methods, and procedures as what is needed in this scenario are not possessed by most public

#### Performance of Entrepreneurial Imaginativeness

We used the quantity and originality of business ideas to measure participants’ performance of entrepreneurial imaginativeness. Consistent with prior research that has utilized raters to evaluate idea originality, the four-point originality scale ranges from 1 (common, mundane, or boring business idea) to 4 (rare, unusual, ingenious, imaginative, or surprising business idea) (Douglas et al., 2006). Three expert raters independently scored the originality of business ideas generated by participants. The overall inter-rater reliability of the idea originality measure was acceptable (Intra-class correlation coefficient (ICC) = 0.673, *p* < 0.001; based on the criteria of the effect size suggested by Cohen et al. (2014), ICC = 0.50 can be considered as a large effect).

#### Entrepreneurial Imaginativeness

We adopted the scale developed by Kier and McMullen (2018) to measure entrepreneurial imaginativeness. Before the formal research, the pre-research was undertaken with 100 participants who met our experimental design. Then, a parsimonious survey measure was created through the confirmatory factor analysis that eliminated any problematic items. The resulting nine-item survey measure was rated on a seven-point Likert-type scale ranging from 1 (strongly disagree) to 7 (strongly agree).

Our final three-factor measurement model showed excellent goodness of fit: Chi-Squared/Degree of freedom (χ^2^/df) = 0.826, Goodness-of-fit index (GFI) = 0.981, Comparative fit index (CFI) = 1, Incremental fit index (IFI) = 1.003, Root mean square error of approximation (RMSEA) = 0, *p* = 0.707. The measure possessed strong reliability (0.832, 0.877, and 0.890 for creative, social, and practical imaginativeness, respectively), convergent validity, and discriminant validity.

### Procedure

In the beginning, participants were randomly assigned to three groups, namely, smart city, smart factory, and no-scenario. Then, participants of the smart city group and smart factory group watched a 5-min video on the theme of smart city or smart factory. After watching a video, they were asked to generate as many business ideas as possible for 5G technology applications in a smart city or smart factory. However, participants of the no-scenario group were directly asked to generate as many business ideas as possible for 5G technology application without watching a video. Subsequently, participants were asked to choose one of the most creative ideas and write a short description of it. The instructions of the experiment and introduction of 5G technology are also given. Finally, participants were asked to fill out a questionnaire about entrepreneurial imaginativeness. All the tasks were completed online.

### Results

In this experiment, we wanted the manipulation of scenarios to induce different performances of entrepreneurial imaginativeness (including the quantity and originality of business ideas). Every participant should be ensured to attend closely to the manipulation and conceive business ideas effortfully. So, based on the suggestions of Hauser et al. (2018), we did not do a manipulation check before this experiment in order for “the sequence of events to capture the participant’s attention and to unfold naturally, and the measures to seem natural and appropriate in the context of the participant’s experience.”

#### Testing Hypothesis 1

We conducted an analysis of covariance (ANCOVA) to examine the differences in performances of entrepreneurial imaginativeness among three scenario setting groups. The independent variable is a *scenario*, the dependent variables are the *quantity* and *originality* of business ideas.

In addition, we used Pearson correlation coefficients to test any significant correlations between the dependent variable (i.e., *quantity* and *originality*) and potential factors (i.e., *entrepreneurial imaginativeness* and *Effort^1^* on the tasks), which determined the covariates to be included in the ANCOVA. The correlations among the variables are described in Table 2. *Quantity* was significantly correlated with *practical imaginativeness* (*r* = 0.197, *p* = 0.003), *Effort 1* (*r* = 0.246, *p* < 0.001), and *Effort 2* (*r* = 0.437, *p* < 0.001); *originality* was significantly correlated with *creative imaginativeness* (*r* = 0.178, *p* = 0.008), *Effort 1* (*r* = 0.291, *p* < 0.001), and *Effort 2* (*r* = 0.45, *p* < 0.001). Although *social imaginativeness* did not have significant correlations with *quantity* and *originality*, it was significantly correlated with *creative imaginativeness* (*r* = 0.378, *p* < 0.001), *practical imaginativeness* (*r* = 0.460, *p* < 0.001), and *Effort 2* (*r* = 0.161, *p* = 0.017). Therefore, five potential factors (i.e., *creative imaginativeness*, *social imaginativeness*, *practical imaginativeness*, *Effort 1*, and *Effort 2*) were used as the covariates to exclude any preexisting differences in participants’ performance of entrepreneurial imaginativeness (i.e., quantity and originality).

**TABLE 2:** Summary of correlations.

	Creative_Ima	Social_Ima	Practical_Ima	Scenario	Effort 1	Effort 2	Quantity	Originality
Creative_Ima	1	2						
Social_Ima	0.378***	1						
Practical_Ima	0.440***	0.460***	1					
Scenario	0.178**	0.083	0.187**	1				
Effort 1	0.056	0.044	0.024	0.254***	1			
Effort 2	0.266***	0.161*	0.216**	0.295***	0.562***	1		
Quantity	0.100	0.040	0.197**	0.259***	0.246***	0.437***	1	
Originality	0.178**	0.081	0.125	0.167*	0.291***	0.450***	0.258***	1

***p < 0.001; **p < 0.01; *p < 0.05 (two-tailed).

*Creative_Ima, creative imaginativeness; Social_Ima, social imaginativeness; Practical_Ima, practical imaginativeness.*

The ANCOVA results indicated that there were significant differences among three groups in *quantity* [*F*(2, 216) = 17.488, *p* < 0.001, η*_*p*_^2^* = 0.142] and originality [*F*(2, 216) = 7.685, *p* = 0.001, η*_*p*_^2^* = 0.068] (Table 3). The results of pairwise comparisons showed that (1) the mean *quantity* of the smart city group was significantly higher than the smart factory group (MD = 0.974, *p* < 0.001); and (2) the mean of *originality* of the smart city group was significantly higher than the smart factory group (*MD* = 0.235, *p* = 0.002), which suggested that the performance of entrepreneurial imaginativeness in the smart city scenario was better in the smart factory scenario (Table 4). Therefore, Hypothesis 1 was supported.

**TABLE 3:** The ANCOVA results of the impact of scenario on quantity and originality.

Dependent variable	Scenario	N	Mean	SD	F	Sig.	η*_*p*_^2^*
Quantity	No-scenario	73	2.110	1.087	17.488	0.000	0.142
	Smart factory	71	1.845	0.936			
	Smart city	75	2.853	1.291			
Originality	No-scenario	73	2.251	0.474	7.685	0.001	0.068
	Smart factory	71	2.188	0.481			
	Smart city	75	2.436	0.383			

**TABLE 4:** Pairwise comparisons of quantity and originality between different scenarios.

Dependent variable	Scenario (I)	Scenario (J)	M.D. (I–J)	Std. error	Sig.	95% confidence interval for difference	
						Lower bound	Upper bound
Quantity	Smart city	No-scenario	0.311	0.176	0.234	–0.113	0.735
	Smart city	Smart factory	0.974*	0.167	0.000	0.057	1.377
	No-scenario	Smart factory	0.663*	0.179	0.001	0.232	1.094
Originality	Smart city	No-scenario	0.005	0.070	1.000	–0.165	0.174
	Smart city	Smart factory	0.235*	0.067	0.002	0.074	0.397
	No-scenario	Smart factory	0.231*	0.072	0.004	0.058	0.403

*Based on estimated marginal means.*

*The mean difference is significant at the 0.05 level.

*MD, mean difference.*

Compared with the no-scenario group, (1) the mean *quantity* of the smart city group was not significantly higher than the no-scenario group (*MD* = 0.311, *p* = 0.234), while the mean *quantity* of the smart factory group was significantly lower than the no-scenario group (*MD* = –0.663, *p* = 0.001); and (2) the mean of *originality* of the smart city group was not significantly higher than the no-scenario group (*MD* = 0.05, *p* = 1.000), while the mean of *originality* of the smart factory group was significantly lower than the no-scenario group (*MD* = –0.231, *p* = 0.004) (Table 4). The results of comparisons with the no-scenario group showed that the familiar scenario (i.e., smart city) did not promote the exertion of entrepreneurial imaginativeness significantly, but the unfamiliar scenario (i.e., smart factory) restrained it.

#### Testing Hypothesis 2

We calculated medians of participants’ entrepreneurial imaginativeness (including creative, social, and practical imaginativeness) scores based on the questionnaires they filled out (Table 5). Then, participants were divided into two levels by median, that is, high and low entrepreneurial imaginativeness. Specifically, there were 115 participants with high creative imaginativeness (HCI) (43 in the smart city group, 38 in the smart factory group, and 34 in the no-scenario group); 104 with low creative imaginativeness (LCI) (32 in the smart city group, 33 in the smart factory group, and 39 in the no-scenario group); 93 with high social imaginativeness (HSI) (38 in the smart city group, 23 in the smart factory group, and 32 in the no-scenario group); 126 with low social imaginativeness (LSI) (37 in the smart city group, 48 in the smart factory group, and 41 in the no-scenario group); 132 with high practical imaginativeness (HPI) (48 in the smart city group, 43 in the smart factory group, and 41 in the no-scenario group); 87 with low practical imaginativeness (LPI) (27 in the smart city group, 28 in the smart factory group, and 32 in the no-scenario group) (Table 6).

**TABLE 5:** Descriptive statistics of entrepreneurial imaginativeness.

Imaginativeness	N	Mean	Median	SD	Minimum	Maximum
Creative_Ima	219	14.685	15	3.461	4	21
Social_Ima	219	15.785	16	2.922	6	21
Practical_Ima	219	14.973	15	3.039	3	21

**TABLE 6:** Distribution of high and low entrepreneurial imaginativeness levels of different scenario setting groups.

Group	LCI (N)	HCI (N)	LSI (N)	HSI (N)	LPI (N)	HPI (N)
Smart city	32	43	37	38	27	48
Smart factory	33	38	48	23	28	43
No-scenario	39	34	41	32	32	41
Total	104	115	126	93	87	132

In addition, when conducting ANCOVA of the impact of *scenario* on the imaginativeness performance of participants with one type of entrepreneurial imaginativeness, we controlled for the other two types of entrepreneurial imaginativeness, *Effort 1* and *Effort 2*.

##### High Entrepreneurial Imaginativeness

The ANCOVA results of the impact of *scenario* on the imaginativeness performance of participants with HCI indicated significant differences in both *quantity* [*F*(2, 112) = 8.846, *p* < 0.001, η*_*p*_^2^* = 0.142] and *originality* [*F*(2, 112) = 4.243, *p* = 0.017, η*_*p*_^2^* = 0.073] (Table 7). The results of pairwise comparisons showed that (1) the mean of *quantity* in the smart city group was significantly higher than that in the smart factory group (*MD* = 1.021, *p* < 0.001), but was not significantly higher than that in the no-scenario group (*MD* = 0.234, *p* = 1.000); (2) the mean of *quantity* in the smart factory group was significantly lower than that in the no-scenario group (*MD* = –0.787, *p* = 0.015); (3) the mean of *originality* in the smart city group was significantly higher than that in the smart factory group (*MD* = 0.229, *p* = 0.027), but was not significantly higher than that in the no-scenario group (*MD* = 0.010, *p* = 1.000); and (4) the mean of *originality* in the smart factory group was not significantly lower than that in the no-scenario group (*MD* = –0.219, *p* = 0.066) (Table 8).

**TABLE 7:** The ANCOVA results of the impact of scenario on quantity/originality (high imaginativeness level).

Imaginativeness level	Imaginativeness performance	Group	N	Mean	SD	F	Sig.	η*_*p*_^2^*
HCI	Quantity	No-scenario	34	2.350	1.178	8.846	0.000	0.142
		Smart factory	38	1.890	1.034			
		Smart city	43	2.910	1.324			
	Originality	No-scenario	34	2.363	0.452	4.243	0.017	0.073
		Smart factory	38	2.219	0.419			
		Smart city	43	2.473	0.365			
HSI	Quantity	No-scenario	32	2.219	0.975	9.251	0.000	0.179
		Smart factory	23	1.739	0.619			
		Smart city	38	2.868	1.379			
	Originality	No-scenario	32	2.365	0.435	3.929	0.023	0.085
		Smart factory	23	2.159	0.437			
		Smart city	38	2.404	0.396			
HPI	Quantity	No-scenario	41	2.439	1.050	15.540	0.000	0.200
		Smart factory	43	1.837	0.785			
		Smart city	48	2.854	1.353			
	Originality	No-scenario	41	2.293	0.429	4.879	0.009	0.073
		Smart factory	43	2.233	0.491			
		Smart city	48	2.424	0.381			

**TABLE 8:** Pairwise comparisons of quantity/originality between different groups (high imaginativeness level).

Imaginativeness level	Imaginativeness performance	Group (I)	Group (J)	MD (I–J)	Std. error	Sig.*^a^*	95% confidence interval for difference*^a^*	
							Lower bound	Upper bound
HCI	Quantity	Smart city	No-scenario	0.234	0.268	1.000	–0.418	0.886
		Smart city	Smart factory	1.021*	0.251	0.000	0.412	1.631
		No-scenario	Smart factory	0.787*	0.274	0.015	0.121	1.453
	Originality	Smart city	No-scenario	0.010	0.092	1.000	–0.214	0.235
		Smart city	Smart factory	0.229*	0.086	0.027	0.019	0.439
		No-scenario	Smart factory	0.219	0.094	0.066	–0.010	0.448
HSI	Quantity	Smart city	No-scenario	0.289	0.265	0.838	–0.359	0.937
		Smart city	Smart factory	1.127*	0.266	0.000	0.479	1.776
		No-scenario	Smart factory	0.838*	0.289	0.014	0.132	1.544
	Originality	Smart city	No-scenario	–0.025	0.104	1.000	–0.279	0.229
		Smart city	Smart factory	0.255*	0.104	0.049	0.001	0.508
		No-scenario	Smart factory	0.280*	0.113	0.046	0.004	0.556
HPI	Quantity	Smart city	No-scenario	0.135	0.227	1.000	–0.416	0.686
		Smart city	Smart factory	1.136*	0.217	0.000	0.609	1.664
		No-scenario	Smart factory	1.001*	0.234	0.000	0.432	1.570
	Originality	Smart city	No-scenario	0.018	0.089	1.000	–0.198	0.234
		Smart city	Smart factory	0.245*	0.085	0.014	0.039	0.452
		No-scenario	Smart factory	0.228*	0.092	0.044	0.005	0.451

*Based on estimated marginal means.*

*The mean difference is significant at the 0.05 level.

*^a^Adjustment for multiple comparisons: Bonferroni.*

The ANCOVA results of the impact of *scenario* on the imaginativeness performance of participants with HSI indicated significant differences in both *quantity* [*F*(2, 90) = 9.251, *p* < 0.001, η*_*p*_^2^* = 0.179] and *originality* [*F*(2, 90) = 3.929, *p* = 0.023, η*_*p*_^2^* = 0.085] (Table 7). The results of pairwise comparisons showed that (1) the mean of *quantity* in the smart city group was significantly higher than that in the smart factory group (*MD* = 1.127, *p* < 0.001), but was not significantly higher than that in the no-scenario group (*MD* = 0.289, *p* = 0.838); (2) the mean of *quantity* in the smart factory group was significantly lower than that in the no-scenario group (*MD* = –0.838, *p* = 0.014); (3) the mean of *originality* in the smart city group was significantly higher than that in the smart factory group (*MD* = 0.255, *p* = 0.049), but was not significantly lower than that in the no-scenario group (*MD* = –0.025, *p* = 1.000); and (4) the mean of *originality* in the smart factory group was significantly lower than that in the no-scenario group (*MD* = –0.280, *p* = 0.046) (Table 8).

The ANCOVA results of the impact of *scenario* on the imaginativeness performance of participants with HPI indicated significant differences in both *quantity* [*F*(2, 129) = 15.540, *p* < 0.001, η*_*p*_^2^* = 0.200] and *originality* [*F*(2, 129) = 4.879, *p* = 0.009, η*_*p*_^2^* = 0.073] (Table 7). The results of pairwise comparisons showed that (1) the mean of *quantity* in the smart city group was significantly higher than that in the smart factory group (*MD* = 1.136, *p* < 0.001), but was not significantly higher than that in the no-scenario group (*MD* = 0.135, *p* = 1.000); (2) the mean of *quantity* in the smart factory group was significantly lower than that in the no-scenario group (*MD* = –1.001, *p* < 0.001); (3) the mean of *originality* in the smart city group was significantly higher than that in the smart factory group (*MD* = 0.245, *p* = 0.014), but was not significantly higher than that in the no-scenario group (*MD* = 0.018, *p* = 1.000); and (4) the mean of *originality* in the smart factory group was significantly lower than that in the no-scenario group (*MD* = –0.228, *p* = 0.044) (Table 8).

From the above results, it can be seen that to the participants with high entrepreneurial imaginativeness (including creative, social, and practical imaginativeness), (1) the smart city group generated more business ideas than the smart factory group and (2) the business ideas generated by the smart city group were more original than the smart factory group. That is, to the participants with high entrepreneurial imaginativeness, their performance of entrepreneurial imaginativeness in a familiar scenario is better than that in an unfamiliar scenario. Therefore, Hypothesis 2a was supported fully. In addition, compared to the no-scenario group, (1) the smart city group did not generate a greater number of more original business ideas, and (2) the smart factory group generated fewer business ideas. It is worth noting that (1) the *originality* of business ideas generated by the participants with HCI was not significantly different between the smart factory group and the no-scenario group, but (2) the *originality* of business ideas generated by the participants with high social/practical imaginativeness in the smart factory group was significantly lower than that in the no-scenario group.

##### Low Entrepreneurial Imaginativeness

The ANCOVA results of the impact of *scenario* on the imaginativeness performance of participants with LCI indicated significant differences in both *quantity* [*F*(2, 101) = 9.354, *p* < 0.001, η*_*p*_^2^* = 0.163] and *originality* [*F*(2, 101) = 3.299, *p* = 0.041, η*_*p*_^2^* = 0.064] (Table 9). The results of pairwise comparisons showed that (1) the mean of *quantity* in the smart city group was significantly higher than that in the smart factory group (*MD* = 0.959, *p* < 0.001), but was not significantly higher than that in the no-scenario group (*MD* = 0.451, *p* = 0.162); (2) the mean of *quantity* in the smart factory group was not significantly lower than that in the no-scenario group (*MD* = –0.508, *p* = 0.100); (3) the mean of *originality* in the smart city group was not significantly higher than that in the smart factory group (*MD* = 0.254, *p* = 0.060) and that in the no-scenario group (*MD* = 0.024, *p* = 1.000); and (4) the mean of *originality* in the smart factory group was not significantly lower than that in the no-scenario group (*MD* = -0.230, *p* = 0.137) (Table 10).

**TABLE 9:** The ANCOVA results of the impact of scenario on quantity/originality (low imaginativeness level).

Imaginativeness level	Imaginativeness performance	Group	N	Mean	SD	F	Sig.	η*_*p*_^2^*
LCI	Quantity	No-scenario	39	1.897	0.968	9.354	0.000	0.163
		smart factory	33	1.788	0.820			
		Smart city	32	2.781	1.263			
	Originality	No-scenario	39	2.154	0.477	3.299	0.041	0.064
		smart factory	33	2.152	0.547			
		Smart city	32	2.385	0.407			
LSI	Quantity	No-scenario	41	2.024	1.172	8.020	0.001	0.120
		smart factory	48	1.896	1.057			
		Smart city	37	2.838	1.214			
	Originality	No-scenario	41	2.163	0.489	5.226	0.007	0.081
		smart factory	48	2.201	0.504			
		Smart city	37	2.468	0.372			
LPI	Quantity	No-scenario	32	1.688	0.998	4.289	0.017	0.098
		smart factory	28	1.857	1.145			
		Smart city	27	2.852	1.199			
	Originality	No-scenario	32	2.198	0.528	3.700	0.029	0.086
		smart factory	28	2.119	0.464			
		Smart city	27	2.457	0.394			

**TABLE 10:** Pairwise comparisons of quantity/originality between different groups (low imaginativeness level).

Imaginativeness level	Imaginativeness performance	Group (I)	Group (J)	MD (I–J)	Std. error	Sig.*^a^*	95% confidence interval for difference*^a^*	
							Lower bound	Upper bound
LCI	Quantity	Smart city	No-scenario	0.451	0.231	0.162	–0.112	1.015
		Smart city	Smart factory	0.959*	0.222	0.000	0.419	1.499
		No-scenario	Smart factory	0.508	0.235	0.100	–0.065	1.081
	Originality	Smart city	no-scenario	0.024	0.112	1.000	–0.249	0.297
		Smart city	Smart factory	0.254	0.107	0.060	–0.007	0.516
		No-scenario	Smart factory	0.230	0.114	0.137	–0.047	0.508
LSI	Quantity	Smart city	No-scenario	0.333	0.246	0.536	–0.264	0.929
		Smart city	Smart factory	0.889*	0.226	0.000	0.340	1.438
		No-scenario	Smart factory	0.557	0.235	0.058	–0.014	1.127
	Originality	Smart city	No-scenario	0.089	0.097	1.000	–0.146	0.324
		Smart city	Smart factory	0.279*	0.089	0.006	0.063	0.495
		No-scenario	Smart factory	0.190	0.092	0.127	–0.035	0.414
LPI	Quantity	Smart city	No-scenario	0.584	0.289	0.140	–0.122	1.290
		Smart city	Smart factory	0.763*	0.267	0.017	0.109	1.417
		No-scenario	Smart factory	0.179	0.276	1.000	–0.497	0.855
	Originality	Smart city	No-scenario	–0.030	0.119	1.000	–0.321	0.262
		Smart city	Smart factory	0.244	0.110	0.091	–0.026	0.514
		No-scenario	Smart factory	0.273	0.114	0.057	–0.006	0.553

*Based on estimated marginal means.*

*The mean difference is significant at the 0.05 level.

*^a^Adjustment for multiple comparisons: Bonferroni.*

The ANCOVA results of the impact of *scenario* on the imaginativeness performance of participants with LSI indicated significant differences in both *quantity* [*F*(2, 123) = 8.020, *p* = 0.001, η*_*p*_^2^* = 0.120] and *originality* [*F*(2, 123) = 5.226, *p* = 0.007, η*_*p*_^2^* = 0.081] (Table 9). The results of pairwise comparisons showed that (1) the mean of *quantity* in the smart city group was significantly higher than that in the smart factory group (*MD* = 0.889, *p* < 0.001), but was not significantly higher than that in the no-scenario group (*MD* = 0.333, *p* = 0.536); (2) the mean of *quantity* in the smart factory group was not significantly lower than that in the no-scenario group (*MD* = –0.557, *p* = 0.058); (3) the mean of *originality* in the smart city group was significantly higher than that in the smart factory group (*MD* = 0.279, *p* = 0.006), but was not significantly higher than that in the no-scenario group (*MD* = 0.089, *p* = 1.000); and (4) the mean of *originality* in the smart factory group was not significantly lower than that in the no-scenario group (*MD* = -0.190, *p* = 0.127) (Table 10).

The ANCOVA results of the impact of *scenario* on the imaginativeness performance of participants with LPI indicated significant differences in both *quantity* [*F*(2, 84) = 4.289, *p* = 0.017, η*_*p*_^2^* = 0.098] and *originality* [*F*(2, 84) = 3.700, *p* = 0.029, η*_*p*_^2^* = 0.086] (Table 9). The results of pairwise comparisons showed that (1) the mean of *quantity* in the smart city group was significantly higher than that in the smart factory group (*MD* = 0.763, *p* = 0.017), but was not significantly higher than that in the no-scenario group (*MD* = 0.584, *p* = 0.140); (2) the mean of *quantity* in the smart factory group was not significantly lower than that in the no-scenario group (*MD* = –0.179, *p* = 1.000); (3) the mean of *originality* in the smart city group was not significantly higher than that in the smart factory group (*MD* = 0.244, *p* = 0.091), and not significantly lower than that in the no-scenario group (*MD* = –0.030, *p* = 1.000); and (4) the mean of *originality* in the smart factory group was not significantly lower than that in the no-scenario group (*MD* = –0.273, *p* = 0.057) (Table 10).

From the above results, it can be seen that the participants with low entrepreneurial imaginativeness (including creative, social, and practical imaginativeness) in the smart city group generated more business ideas than those in the smart factory group. Only the business ideas created by the participants with LSI in the smart city group were more original than those in the smart factory group. There were no significant differences in the *originality* of business ideas between the participants with low creative or practical imaginativeness in the smart city group and that in the smart factory group. Moreover, compared to the participants with low entrepreneurial imaginativeness in the no-scenario group, there were no significant differences in the *quantity* and *originality* of business ideas between them and the smart city group/the smart factory group. Therefore, Hypothesis 2b was partially supported.

## General Discussion

Entrepreneurial imaginativeness is the transformation of a person’s inner cognitive abilities into explicit new venture ideas. It is a visualization process in one’s brain that combines captured information and previous professional knowledge and experience with the latent ability of imagination (McMullen and Kier, 2017). The visualization of a new venture idea is realized through the construction of a scenario in which a specific demand is found and met. Although video scenarios have been confirmed to support imagination and favor team cooperation in the process of creating business ideas (Pillan et al., 2014), scenarios were closely related to daily life in prior studies; that is, these scenarios were familiar to participants and beneficial to the utilization of their prior knowledge, expertise, and experience. However, findings from such studies have not distinguished the impacts on the exerting of imaginativeness in familiar scenarios and unfamiliar scenarios. Nor have they confirmed that scenarios with different levels of public familiarity have the same or different effect(s) on the generation of new ideas for people with different levels of imaginativeness. An exploratory response to these two research questions is offered in this study.

First, the performance of entrepreneurial imaginativeness of the smart city group was significantly better than the smart factory group. However, compared to the no-scenario group, the smart city group did not have a significant advantage on the performance of entrepreneurial imaginativeness, and the smart factory group performed obviously worse. This result indicated that an unfamiliar scenario limited the effective utilization of participants’ previous knowledge and experience, resulting in the insufficient exertion of entrepreneurial imaginativeness. In addition, we compared high-frequency words of new venture ideas between the smart city group and the no-scenario groups and found that ranking in the top three, *smart traffic* (including *vehicle-road synergy* and a*utopilot*) appeared 56 times in the smart city group and 28 times in the no-scenario group, *telemedicine* (including *remote surgery* and *remote consultation*) appeared 36 times in the smart city group and 29 times in the no-scenario group, and *virtual reality*/*augmented reality* appeared 21 times in the smart city group and 33 times in the no-scenario group. The analysis of high-frequency words showed that participants still selected the scenarios they were familiar with to apply a new technology when no scenarios were given. This explained why the difference in entrepreneurial imaginativeness performances between the smart city group and the no-scenario group was not statistically significant.

Second, the participants with high entrepreneurial imaginativeness (including creative, social, and practical imaginativeness) in the smart city group outperformed significantly the participants with high entrepreneurial imaginativeness in the smart factory group. That is, the abilities and experience of the participants with high entrepreneurial imaginativeness were brought to full use in a familiar scenario to create new venture ideas. However, the impacts of an unfamiliar scenario on the exertion of high entrepreneurial imaginativeness were complex. Specifically, the HCI that makes novel connections to form new means-ends relationships (Eckhardt and Shane, 2003) was not completely inhibited by an unfamiliar scenario and still benefit to generate business ideas with good originality; but the high social and practical imaginativeness were negatively influenced by an unfamiliar scenario, resulting in the failure of this two imaginativeness to play out through the intentional and knowledge-based empathy.

Third, compared with the participants with low entrepreneurial imaginativeness (including creative, social, and practical imaginativeness) in the smart factory group, the participants with low entrepreneurial imaginativeness in the smart city group only had an advantage on the quantity of business ideas. That is, to the participants with low entrepreneurial imaginativeness, a familiar scenario still played a more active role in facilitating their entrepreneurial imaginativeness to create more business ideas than an unfamiliar scenario. However, the scenario is only an external factor acting on the imaginative process, which cannot fundamentally enhance the level of the idea originality. Especially for the participants with low creative and practical imaginativeness, even in a familiar scenario, they cannot create business ideas with more originality.

In summary, this study revealed that a familiar scenario did indeed inspire entrepreneurial imaginativeness more than an unfamiliar scenario, especially for high entrepreneurial imaginativeness. These results further clarified that the scenarios that provided a familiar space–time context for empathy simulations were tools to support imagination (Pillan et al., 2014), providing a new respective for understanding the relationship between empathy process and entrepreneurial opportunity recognition and evaluation processes (Packard and Burnham, 2021). Moreover, this study introduced scenarios into the external factors that inspire entrepreneurial imaginativeness, further deepening the research of entrepreneurial imaginativeness (Kier and McMullen, 2018). Meanwhile, this study had practical implications for entrepreneurial practice, especially those that make human life better based on new digital technologies.

In addition, we suggest that individuals utilize different familiar scenarios to foster their entrepreneurial imaginativeness and team leaders improve team performances of entrepreneurial imaginativeness by selecting members who are not only familiar with the task scenarios but also in the high level of entrepreneurial imaginativeness, specifically as follows.

### For Individual

It is recommended that individuals make full use of familiar scenarios to train their divergent thinking, foster their empathy and ability of perspective-taking, and establish and continuously enrich their knowledge system and methodology. For example, (1) generating alternative solutions to meet existing demands in familiar scenarios by making novel connections with old or seemingly unrelated elements; (2) broadening cognitive and knowledge boundaries of divergent thinking by repeatedly comparing the existing solutions to the same or similar demand(s) appearing in different familiar scenarios; (3) enriching feelings and experiences through the transformation of different characters in the same familiar scenario or the comparison of the same characters in different familiar scenarios so as to find out the real demands of people in these scenarios and the reasons why these demands are not been properly satisfied; and (4) summarizing practical problem-solving experience (including knowledge, methods, and know-how) by identifying and classifying the commonalities and differences in different familiar scenarios to make their knowledge system and methodology appropriate for more scenarios.

### For Team

We suggest that team leaders who need to lead a team through a creative task should do two necessary preparations in advance. First of all, team leaders should select members who are familiar with the task scenario and have high entrepreneurial imaginativeness to ensure that the knowledge, expertise, and experience of the members can serve their creative tasks. Secondly, team leaders should make elaborate arrangements as follows: (1) members with HSI play different roles to understand others’ wants and needs, and further effectively discover demand; (2) members with HCI conduct brainstorming to question existing solutions taken for granted and propose new ideas by creatively using or combining things; and (3) members with HPI deduce all the steps needed to realize these ideas, as well as the prerequisites and tools for each step. Such a task division based on the task scenario can strengthen perceived task interdependence to promote knowledge-sharing and enhance team creativity (Fong et al., 2018).

In the end, a limitation of this study might be that we did not design the interaction of participants with potential users. The reason is that it is difficult to effectively control the uncertainties of the interaction in an experiment. However, entrepreneurial imaginativeness, especially social imaginativeness, requires human interactions to make full use of it. In the future, case studies can be used to explore the influence mechanism of scenarios on entrepreneurial imaginativeness.

## Data Availability Statement

The raw data supporting the conclusions of this article will be made available by the authors, without undue reservation.

## Author Contributions

YC and MW jointly developed this research question and designed the experiment. YC and YL conducted the experiment and completed data collection. YC completed the data analysis and wrote this manuscript. RL participated in the discussion of revising the manuscript and gave modification suggestions. YL completed the English editing and proofreading of the manuscript. MW provided financial support for this research as the leader of two funded projects. All authors contributed to the article and approved the submitted version.

## Conflict of Interest

The authors declare that the research was conducted in the absence of any commercial or financial relationships that could be construed as a potential conflict of interest.

## Publisher’s Note

All claims expressed in this article are solely those of the authors and do not necessarily represent those of their affiliated organizations, or those of the publisher, the editors and the reviewers. Any product that may be evaluated in this article, or claim that may be made by its manufacturer, is not guaranteed or endorsed by the publisher.

## Footnotes

1 We measured *Effort* in two different ways, that is, *Effort 1* is the time spent by a participant to complete the tasks, measured in seconds, while *Effort 2* is the amount of words written to describe ideas. *Effort 1* and *Effort 2* were calculated by a computer program.
