# Supplementary material for: The Impact of Scenarios on the Performance of Entrepreneurial Imaginativeness: Evidence From an Experiment
Source: Front Psychol. 2022 Mar 31;13:813657. doi: 10.3389/fpsyg.2022.813657 (PMC9009533; doi:10.3389/fpsyg.2022.813657)
Supplement: Supplementary file 1 [file Data_Sheet_1.docx]

The impact of scenarios on the performance of entrepreneurial imaginativeness: evidence from an experiment

# APPENDIX A: New Venture Ideation

***Instructions:*** In this section, you will watch a 5-minute video on smart cities^[[1]](#footnote-1)^. After watching it, please write as many business ideas as possible to commercialize Fifth Generation Mobile Communication Technology (5G) in smart city scenarios^[[2]](#footnote-2)^, based on the 5G technology characteristics described below. Then, please select the one you think is the best from your business ideas and describe it, including but not limited to its potential customers, technical implementation, product performance and business model.

**5G Technology Description**

5G is the latest generation of mobile cellular telecommunications system with three significant technical features:

1.Ultra-high speed

The data transmission speed of 5G network is 100 times that of 4G network, up to 10Gbps, allowing users to have a smoother Internet experience and providing more possibilities for new communication methods.

2.Ultra-low latency

5G network latency (end-to-end data transmission time) is reduced to 1 millisecond, equivalent to 1% of the human eye blink time, enabling near real-time request-response interaction, which promotes 5G applications in the fields of high accuracy and/or security.

3. Oversized connection

The connection density of 5G network can reach 1 million per square kilometer, ensuring high-speed data transmission in areas with high population density and frequent demands for use. In addition, the connection object of 5G also includes things, which realizes the intelligent world of "Internet of Everything" and brings more possibilities for the future.

# APPENDIX B: Questionnaire of entrepreneurial imaginativeness

**The scale of entrepreneurial imaginativeness**

***Creative imaginativeness***

A1: I consider myself to be inventive.

A2: I consider myself to be innovative.

A3: I demonstrate originality in my work.

***Social imaginativeness***

B2: I always make an effort to see the world through other people’s eyes.

B3: It is easy for me to understand why people feel the way they do.

B4: I have a good sense for what other people are feeling.

***Practical imaginativeness***

C1: I tend to be good at project management.

C2: I can picture what the bottleneck of a system will be.

C3: Before I face a new situation, I picture the issues I may encounter and plan accordingly.

| **Table Ⅰ Convergent validity: AVE and CR of the measurement model** | | | |
| --- | --- | --- | --- |
| Path | λ | AVE | C.R. |
| A1→A | 0.935 | 0.671 | 0.853 |
| A2→A | 0.920 |  |  |
| A4→A | 0.540 |  |  |
| B2→B | 0.807 | 0.720 | 0.885 |
| B3→B | 0.880 |  |  |
| B4→B | 0.857 |  |  |
| C1→C | 0.837 | 0.745 | 0.898 |
| C2→C | 0.883 |  |  |
| C3→C | 0.869 |  |  |
| Note: The measurement model has been revised based on the results of modification indices.  λ= standardized factor loadings；AVE means the average variance extracted measure；  CR means composite reliability.  A is creative imaginativeness, B is social imaginativeness and C is practical imaginativeness. | | | |

| **Table Ⅱ Discriminant validity：Pearson correlation and SQRT-AVE** | | | |
| --- | --- | --- | --- |
|  | A | B | C |
| A | - |  |  |
| B | .396^**^ | - |  |
| C | .493^**^ | .460^**^ | - |
| SQRT-AVE | 0.819 | 0.849 | 0.863 |
| **. The mean difference is significant at the 0.01 level.  A is creative imaginativeness, B is social imaginativeness and C is practical imaginativeness;  SQRT-AVE is the square root of AVE. | | | |

1. *Note: participants in the smart-factory group are provided a video on smart factories, but participants in the no-scenario group have no video to watch*. [↑](#footnote-ref-1)
2. *Note: participants in the smart-factory group are asked to write business ideas as many as possible to commercialize 5G in smart factory scenarios, while participants in the no-scenario group are permitted to create business ideate to commercialize 5G in any scenario.* [↑](#footnote-ref-2)
